# Supplementary material for: The Importance of Assay Imprecision near the Screen Cutoff for Newborn Screening of Lysosomal Storage Diseases
Source: Int J Neonatal Screen. 2019 Mar 27;5(2):17. doi: 10.3390/ijns5020017 (PMC6641561; doi:10.3390/ijns5020017)
Supplement: Supplementary file 1 [file IJNS-05-00017-s001.pdf]

Supplemental Information

1. Theory of probability distribution functions and their convolution as applied to NBS.

In this article probability distribution functions of the type shown in Figure 1 have been presented. The best known probability distribution function, PDF, is the Normal or Gaussian distribution, $G(z)$, which is a continuous function of the variable z. For the Normal distribution:

$$G(z) = \frac{1}{\sqrt{2\pi}} e^{-\frac{1}{2}z^2}$$

$$z = \frac{(x - \mu)}{\sigma}$$

Here, x is the enzymatic activity, μ and σ are the mean enzymatic activity and standard deviation of the enzymatic activity for the newborn population, respectively. The probability distribution function in terms of x, $F(x)$ is easily related to $G(z)$ as: $F_N(x) = \frac{dz}{dX} G(z)$. In this case $\frac{dz}{dX} = \frac{1}{\sigma}$.

As described in the main text, the Log-Normal probability distribution function is more appropriate for NBS. In this case:

$$z_{LN} = \frac{1}{w} ln\left(\frac{x}{\mu}\right) + \frac{w}{2} \quad \text{where} \quad w^2 = ln\left(1 + \left(\frac{\sigma}{\mu}\right)^2\right)$$

The log-normal probability distribution function in terms of x is:

$$F_{LN}(x) = \frac{dZ_{LN}}{dx} G(Z_{LN}) = \frac{1}{wx} G(Z_{LN})$$

A typical $F_{LN}(x)$ is shown as Figure 1 in the main text. In practice, if $\mu \geq 5\sigma$ it is difficult to distinguish between the normal and the log-normal distributions, and hence unnecessary. This would correspond to the case where the mean enzymatic activity is large enough such that the number of newborns in the population with enzymatic activity close to 0 is very small. Or said another way, skewing of the Gaussian due to fact that x must be a positive number is very small.

In the context of NBS, there are two Log-Normal distributions, one for the healthy newborns, $F_{LN,N}(x)$, and one for the diseased newborns, $F_{LN,D}(x)$. Each distribution has its own mean (μ_N and μ_D) and width (σ_N and σ_D).

The assay of enzymatic activity has a degree of imprecision (also called error), which we call the standard imprecision of the measurement, σ_M. To measure the imprecision, one takes

multiple 3 mm punches from the same dried blood spots and submits each punch to the same assay. This is what the Centers for Disease Control and Prevention (CDC) does for their Quality Control dried blood spots, for which data is provided as certificate reports (https://www.cdc.gov/labstandards/nsqap_resources.html). The measurements are fit to a Gaussian distribution to obtain the mean μ_M and standard error σ_M.

The *observed* PDF (i.e. Figure 1 in the main text) contains variation due to the imprecision and variation in the population due to all other factors. The latter is referred to as the no-*imprecision* PDF. The mathematics of how to combine these two PDFs is well founded and is generally treated as a *convolution*. The appropriate convolutions, $f_N(x)$, for the normal newborns, and the one for the disease newborns, $f_D(x)$, are given below:

$$f_N(x) = \int_0^\infty F_{LN,N}(\chi) \cdot G\left(\frac{x-\chi}{\sigma_M}\right)\frac{1}{\sigma_M}d\chi \text{ and } f_D(x) = \int_0^\infty F_{LN,D}(\chi) \cdot G\left(\frac{x-\chi}{\sigma_M}\right)\frac{1}{\sigma_M}d\chi$$

Note that the variable of integration, χ, runs over all possible values of the enzymatic activity, x, in the dried blood spot. The function *G* in the above equation is the Gaussian function for the imprecision in the measurement. The above two equations for the convolution are based on a single imprecision distribution, wherein the standard error of measurement does not depend on the mean assay value. However, the data shown in Figure 2 in the main text shows that σ_M does vary with μ_M. This is the general case with most measurements in dried blood spots. We can incorporate this dependence in the above convolution equations by noting that, in general, $\sigma_M = \sigma_M(\chi)$. For example, in Figure 2, we show a possible model function that could be used in the above convolution equations. The identity of this model function is not important as long as it well fits the data. The function *G* in the above equation is the Gaussian function for the imprecision in the measurement.

From the distribution of newborn assays, one can obtain the fraction of patients between 0 and any specific assay value, x_s.

$$I(x_s) = \int_{x=0}^{x_s} f(x)dx$$

The fraction, $I(x_s)$, is zero as $x_s \to 0$ and is one as $x_s \to \infty$. The fraction monotonically increases as x_s increases. This applies to both the normal and the disease population:

$$I_N(x_s) = \int_{x=0}^{x_s} f_N(x)dx \quad \text{and} \quad I_D(x_s) = \int_{x=0}^{x_s} f_D(x)dx$$

One would like a clear distinction between the normal and disease population. However, with continuous distributions, this is not possible; there will always be some amount of overlap. In the case of NBS assays, the mean of the normal group is much greater than that of the disease group, $\mu_N \gg \mu_D$. If we consider any specific assay threshold value, x_s, the fraction of the disease

group with a value less than x_s belongs to the truly positive group. The fraction in this group is the true positive rate, TPR, $TPR = I_D(x_s)$. Those in the disease group with a value larger than x_s are in the false negative group, FN_D; and the false negative rate, FNR, is $FNR = FN_D = 1 - I_D(x_s)$ ("false negatives"). The fraction of the normal group with assay values less than x_s, called FP_N, has the false positive rate, FPR, $FPR = FP_N = I_N(x_s)$ ("false positives"), and those with values greater than x_s are truly negative. Therefore, the true negative rate, TNR, is $TNR = 1 - FP_N = 1 - I_N(x_s)$. We assign this value x_s as the screen cutoff, and all of these rates are continuous functions of the screen cutoff. A typical receiver operating characteristic (ROC) plot is a plot of TPR vs FPR.

Because the TPR and FPR are both continuous functions of the screen cutoff (which is not shown or even knowable from the plot), the plot is also called a parametric plot. A plot of this sort emphasizes the sensitivity of the test as a tradeoff with the probability of false alarm. However, for heuristic reasons we choose to plot the FPR as a function of the FNR, which emphasizes the probability of false alarm as a trade off with missing disease positives.

The goal now is twofold: to identify a proper way to distinguish between the two groups and a way to characterize what is an acceptable overlap. As an example: If there are N_N newborns in the normal distribution and N_D newborns in the disease distribution, then the total number of false identifications would be $N_T = N_N \cdot FN_N + N_D \cdot FP_D$. Therefore, if one wished to minimize the total number of false observations (we want N_T to be as small as possible) then that happens when $N_N f_N(x_s) = N_D f_D(x_s)$. Thus, it is possible to uniquely define an x_s that minimizes the total number of falsely placed individuals. Unfortunately this is not the only criterion, but it is an interesting starting point for defining the best cutoff and provides insight into the approximate region where the cutoff probably will be found.

2. Procedure for carrying out the convolutions.

All calculations were done using Octave version 4.4.0. Octave is Matlab compliant. The functions were evaluated as described above, and the convolution integral was done pointwise for each value of x using the trapezoid rule, where the upper limit on the integral was increased until no detectable change in the integral was observed. Generally $x > 20$ was sufficient, and the integral was represented by 5000 intervals. The computer code is available upon request. Contact the authors if you prefer to have your data analyzed by them.

3. The convolution process.

To illustrate the convolution process, we refer to Supplemental Figure 1. Shown is a *no-imprecision* PDF (green curve) as well as an *imprecisio*n PDF (blue curve), with the latter positioned somewhere along the X-axis (i.e. with its mean value at some specific enzymatic activity, x, in this case 5 µmol/h/L). The *imprecision* PDF also is described by the standard deviation of the measurement, σ_M, which determines the width of this PDF. To compute the

convolution PDF at this x value we multiply the value of the *no-imprecision* PDF at some other value x' by the value of the *imprecision* PDF at x' and repeat this pairwise multiplication for all values of x' to cover the full range of enzymatic activities. All products are added together. For values of x' far away from the mean value of the *imprecision* PDF, the contributions to the sum are small because the *imprecision* PDF is close to zero in these regions. To obtain the convolution at some new value of x we move the *imprecision* PDF to the new value along the X-axis and repeat the process again.

Imagine the case where the imprecision PDF has a very small σ_{M}. The products are close to zero except for x' close to the mean value of the *imprecision* PDF, and then the product is close to the value of the *no-imprecision* PDF at the same position. When this is repeated for all values along the X-axis, the convolution is nearly identical to the original *no-imprecision* PDF. However, as σ_M increases the convolution is broader than the *no-imprecision* PDF at the tails and reduced in height near the mean of the *no-imprecision* PDF (since the area of PDFs must be unity).

Supplemental Figure 1. Shown in green is the Log-Normal PDF for the healthy newborns (mean = 10 µmole/h/L and width = 3.8 µmole/h/L). The blue curve is a Normal distribution with mean 5 µmole/h/L and width 0.5 µmole/h/L (a typical imprecision data set carried out with multiple punches of a Quality Control dried blood spot). The blue curve is re-scaled to touch the green curve at 5 µmole/h/L. The black curve is the product of the two PDFs (not scaled). Notice the slight shift to the right for the maximum value along this curve. The area under the black curve is the value of the convolved function for x = 5 µmole/h/L. This process is repeated for all values of x to give the convolution.

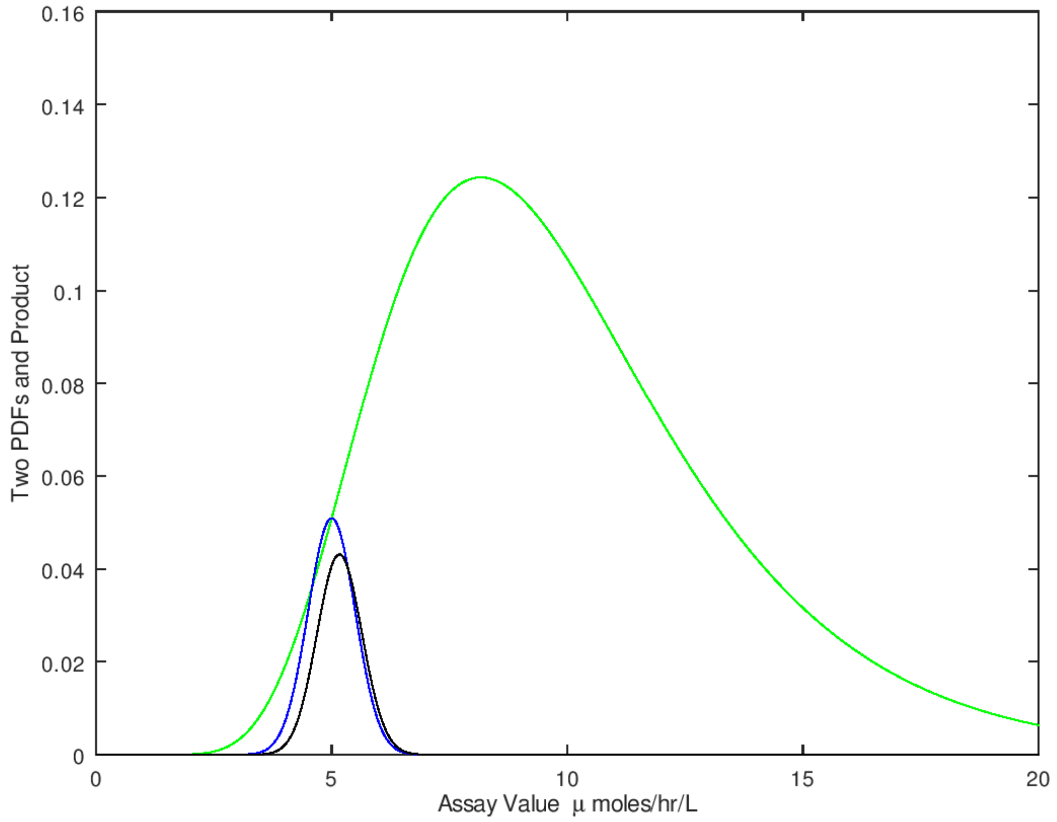

0.16
0.14
0.12
0.1
0.08
0.06
0.04
0.02
0
Two PDFs and Product
0
5
10
15
20
Assay Value μ moles/hr/L
